# Supplementary material for: Phylogenomic, Morphological, and Phylogenetic Evidence Reveals Five New Species and Two New Host Records of Nectriaceae (Hypocreales) from China
Source: Biology (Basel). 2025 Jul 17;14(7):871. doi: 10.3390/biology14070871 (PMC12292661; doi:10.3390/biology14070871)
Supplement: Supplementary file 1 [file biology-14-00871-s001.zip › Supplementary Fig. S1-S5.pdf]

Fig. S 1

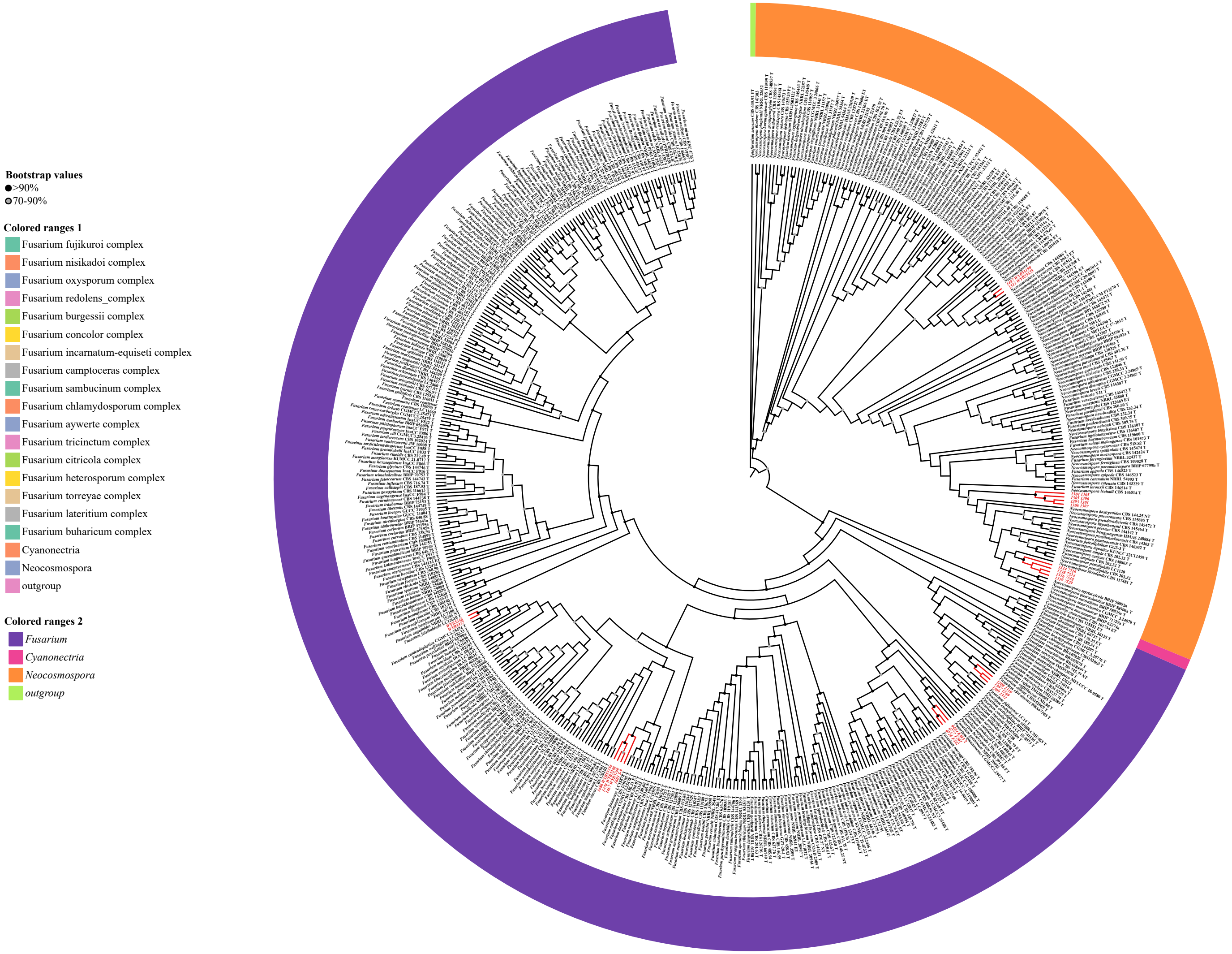

Figure S1: Maximum Likelihood (ML) phylogenetic analyses based on the combined *tef1-rpb1-pb2* gene regions of the *Fusarium* and *Neocosmospora*. *Setofusarium setosum* CBS 635.92 ET was used as an outgroup. Strains isolated in this study were indicated in red. The RAxML boot-strap support values (ML-BS > 70%) were displayed at the nodes (ML-BS).
Ex-type, ex-epitype, and ex-neotype strains were indicated with T, ET, and NT, respectively.

Fig. S 2

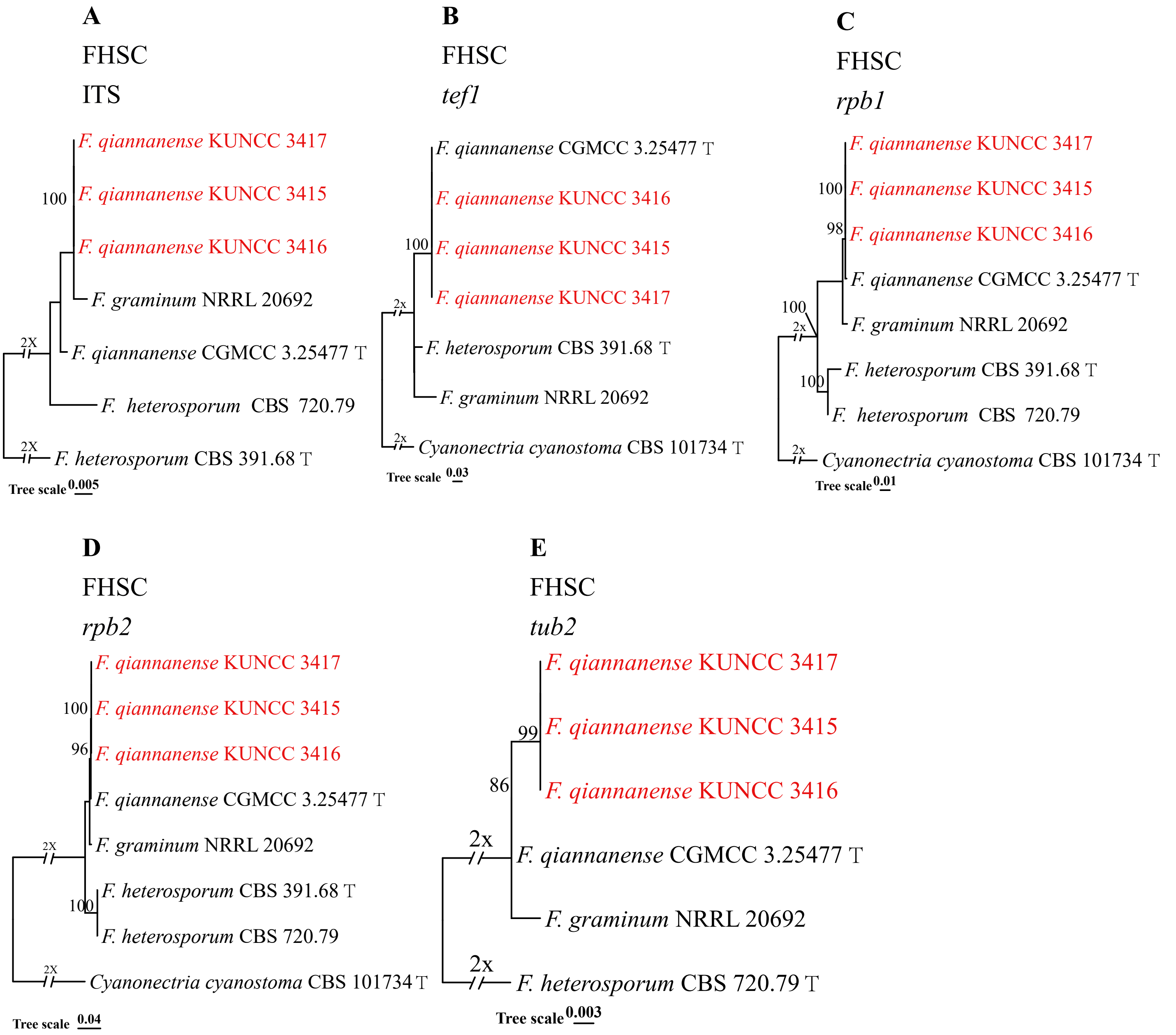

Fig. S2. Phylogeny of the *Fusarium heterosporum* species complex (FHSC) inferred from the ITS (A), *tef*1 (B), *rpb*1 (C), *rpb2* (D), and *tub2* (E) loci, respectively. *Cyanonectria cyanostoma* (CBS 101734 T) served as the outgroup. Strains sequenced in this study are indicated in red. RAxML bootstrap support values (ML-BS ≥ 70%) are shown at the nodes. Ex-type, ex-epitype, and ex-neotype strains are denoted as T, ET, and NT, respectively.

Fig. S 3

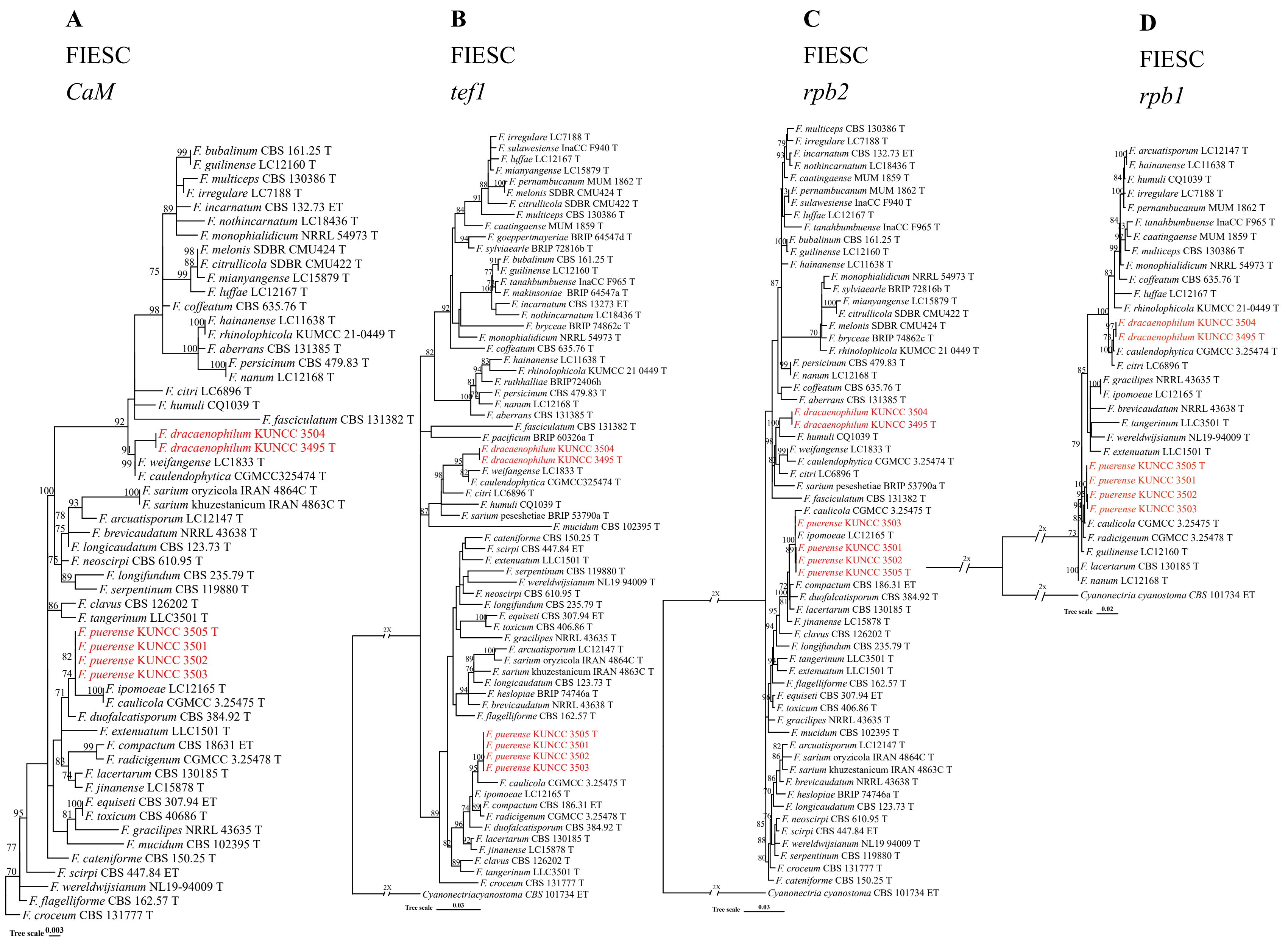

Fig. S3. Phylogeny of the *Fusarium incarnatum-equiseti* species complex (FIESC) inferred from the *CaM* (A), *tef*1 (B), *rpb*2 (C), and *rpb*1 (D) loci, respectively. *Cyanonectria cyanostoma* (CBS 101734 T) served as the outgroup. Strains sequenced in this study are indicated in red. RAxML bootstrap support values (ML-BS ≥ 70%) are shown at the nodes. Ex-type, ex-epitype, and ex-neotype strains are denoted as T, ET, and NT, respectively.

Fig. S 4

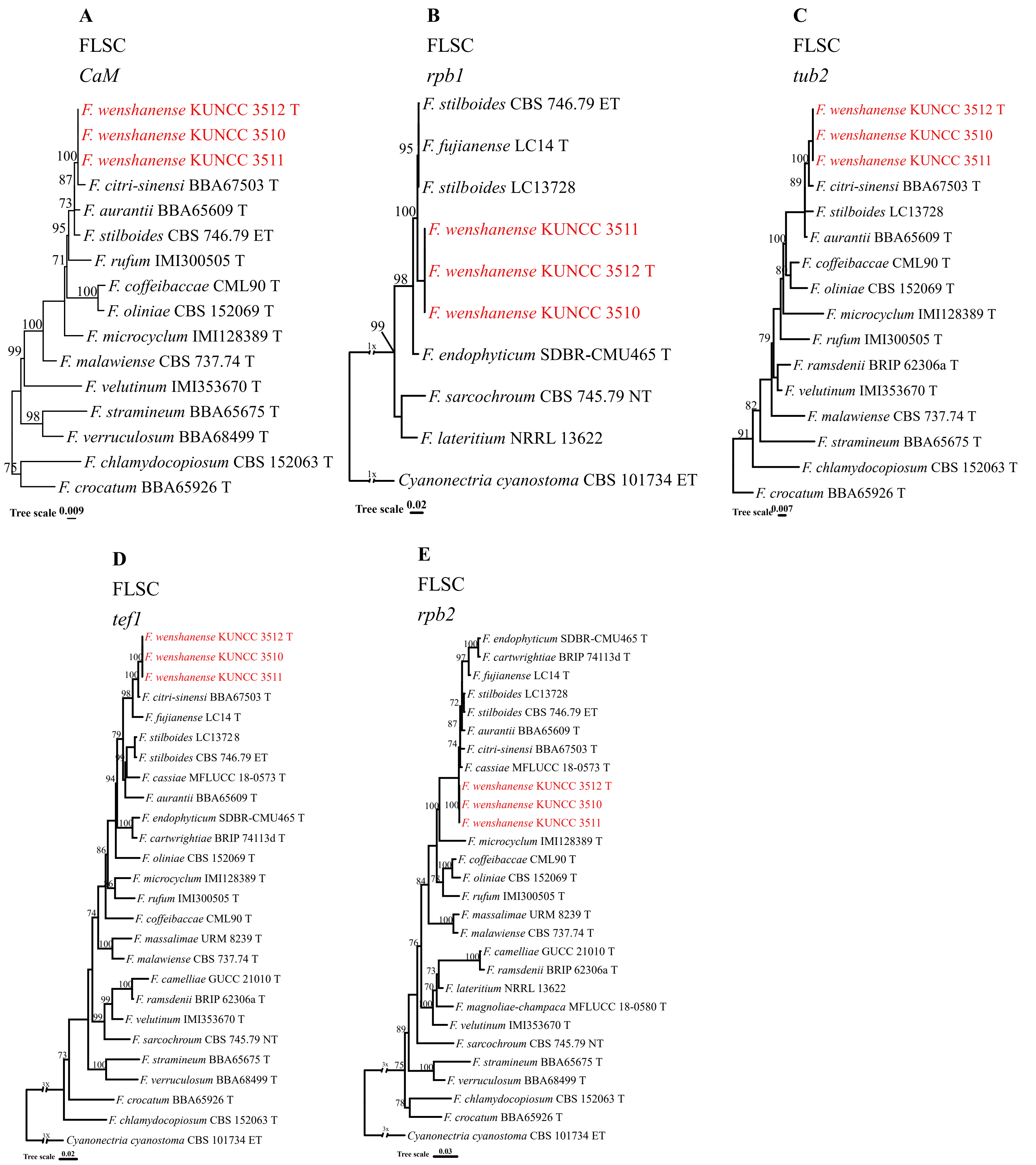

Fig. S4. Phylogeny of the *Fusarium lateritium* species complex (FLSC) inferred from the *CaM* (A), *rpb*1 (B), *tub2* (C), *tef1* (D), and *rpb2* (F) loci, respectively. *Cyanonectria cyanostoma* (CBS 101734 T) served as the outgroup. Strains sequenced in this study are indicated in red. RAxML bootstrap support values (ML-BS ≥ 70%) are shown at the nodes. Ex-type, ex-epitype, and ex-neotype strains are denoted as T, ET, and NT, respectively.

Fig. S 5

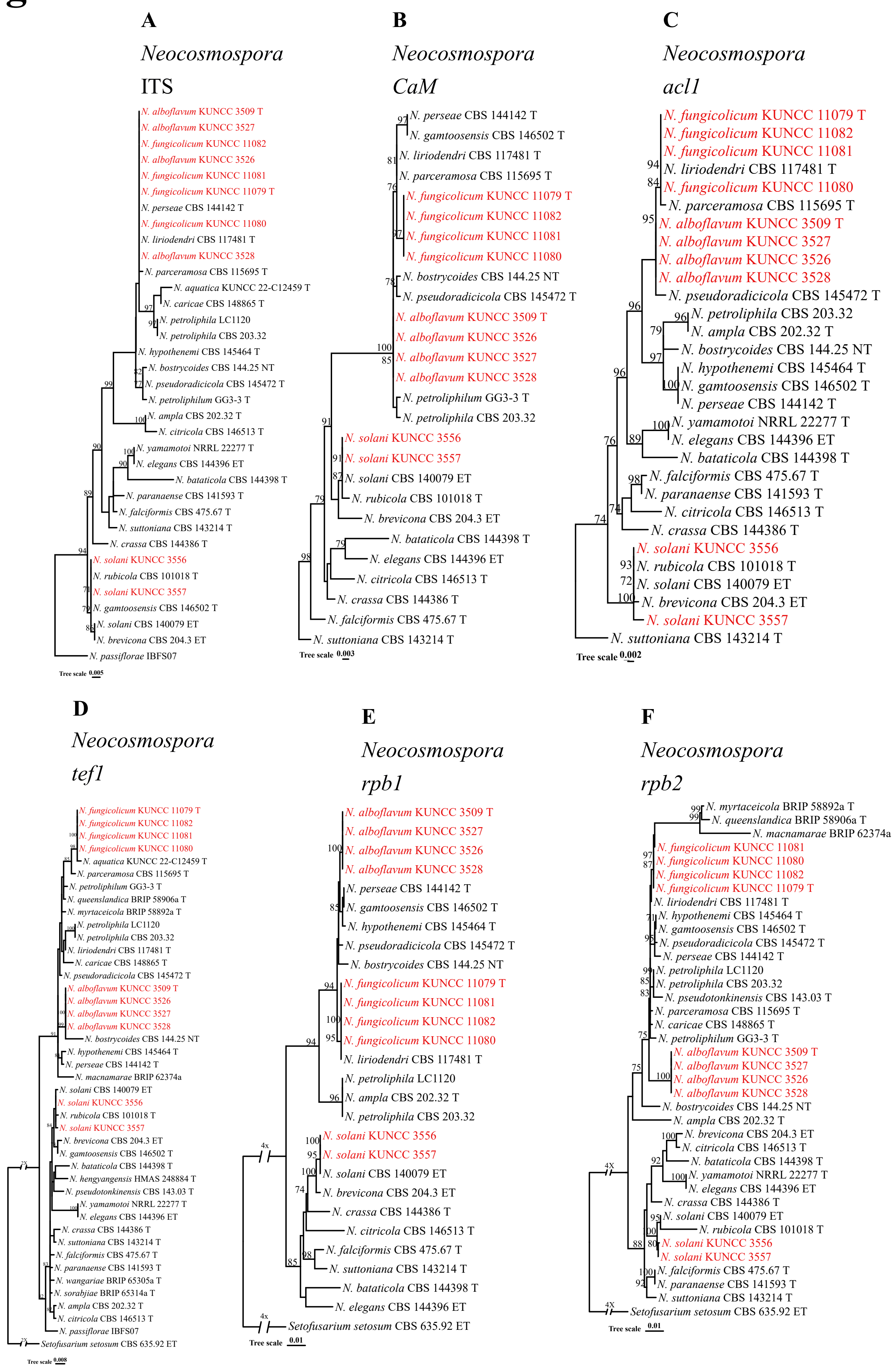

Fig. S5. Phylogeny of the *Neocosmospora* inferred from the ITS (A), *CaM* (B), *acl1* (C), *tef1* (D), *rpb*1 (E), and *rpb2* (F) loci, respectively. *Setofusarium setosum* (CBS 635.92 ET) served as the outgroup. Strains sequenced in this study are indicated in red. RAxML bootstrap support values (ML-BS ≥ 70%) are shown at the nodes. Ex-type, ex-epitype, and ex-neotype strains are denoted as T, ET, and NT, respectively.
