# Supplementary material for: Phylogenomic, Morphological, and Phylogenetic Evidence Reveals Five New Species and Two New Host Records of Nectriaceae (Hypocreales) from China
Source: Biology (Basel). 2025 Jul 17;14(7):871. doi: 10.3390/biology14070871 (PMC12292661; doi:10.3390/biology14070871)
Supplement: Supplementary file 1 [file biology-14-00871-s001.zip › Supplementary Table S1.pdf]

Table S1. Primers information of PCR amplification of *Fusarium* and *Neocosmospora*.

Genes	Abbreviation	Primers	Direction	Sequence (5'/3')	PCR amplification procedures (This study)	Reference
ATP citrate lyase	*acl1*	230up	Forward	AGCCCGATCAGCTCATCAAG	95 °C 4 min; 35 cycles of 95 °C 15 s, 59°C 45 s, 72 °C 60 s; 72 °C 5 min; 10 °C soak	[21]
		1220low	Reverse	CCTGGCAGCAAGATCVAGGAAGT		
Internal transcribed spacer region of the nrDNA	ITS	ITS5	Forward	GGAAGTAAAAGTCGTAACAAGG	95 °C 4 min; 35 cycles of 95 °C 15 s, 55°C 30 s, 72 °C 40s; 72 °C 5 min; 10 °C soak	[93]
		ITS4	Reverse	TCCTCCGCTTATTGATATGC		
Beta-tubulin	*tub2*	T1	Forward	AACATGCGTGAGATTGTAAGT	95 °C 4 min; 35 cycles of 95 °C 15 s, 55 °C 45 s, 72 °C 60 s; 72 °C 10 min; 10 °C soak	[94]
		T2	Reverse	TAGTGACCCTTGGCCCAGTTG		
Calmodulin	*CaM*	CL1	Forward	GARTWCAAGGAGGCCTTCTC	95 °C 3 min; 35 cycles of 95 °C 15 s, 56 °C 30 s, 72 °C 60 s; 72 °C 10 min; 10 °C soak	[95]
		CL2A	Reverse	TTTTTGCATCATGAGTTGGAC		
Translation elongation factor 1-alpha	*tef1*	EF-1	Forward	ATGGGTAAGGARGACAAGAC	95 °C 4 min; 35 cycles of 95 °C 15 s, 55 °C 45 s, 72 °C 60 s; 72 °C 5 min; 10 °C soak	[96]
		EF-2	Reverse	GGARGTACCAGTSATCATG		
RNA polymerase largest subunit	*rpb1*	Fa	Forward	CAYAARGARTCYATGATGGGWC	95 °C 5 min; 35 cycles of 95 °C 15 s, 57 °C 45 s, 72 °C 80 s; 72 °C 10 min; 10 °C soak	[91]
		G2R	Reverse	GTCATYTGDGTDGCDGGYTCDCC		
RNA polymerase second largest subunit	*rpb2*	5f2	Forward	GGGGWGAYCAGAAGAAGGC	95 °C 4 min; 35 cycles of 95 °C 15 s, 57 °C 45 s, 72 °C 80 s; 72 °C 10 min; 10 °C soak	[97]
		11ar	Reverse	GCRTGGATCTTRTCRTCSACC		[98]
